# Supplementary material for: A dual sgRNA library design to probe genetic modifiers using genome-wide CRISPRi screens
Source: BMC Genomics. 2023 Oct 30;24:651. doi: 10.1186/s12864-023-09754-y (PMC10614335; doi:10.1186/s12864-023-09754-y)
Supplement: Supplementary file 1 — Supplementary Material 1 [file 12864_2023_9754_MOESM1_ESM.docx]

**Supplemental File 1**

**Supplemental Figures and Figure Legends**

**
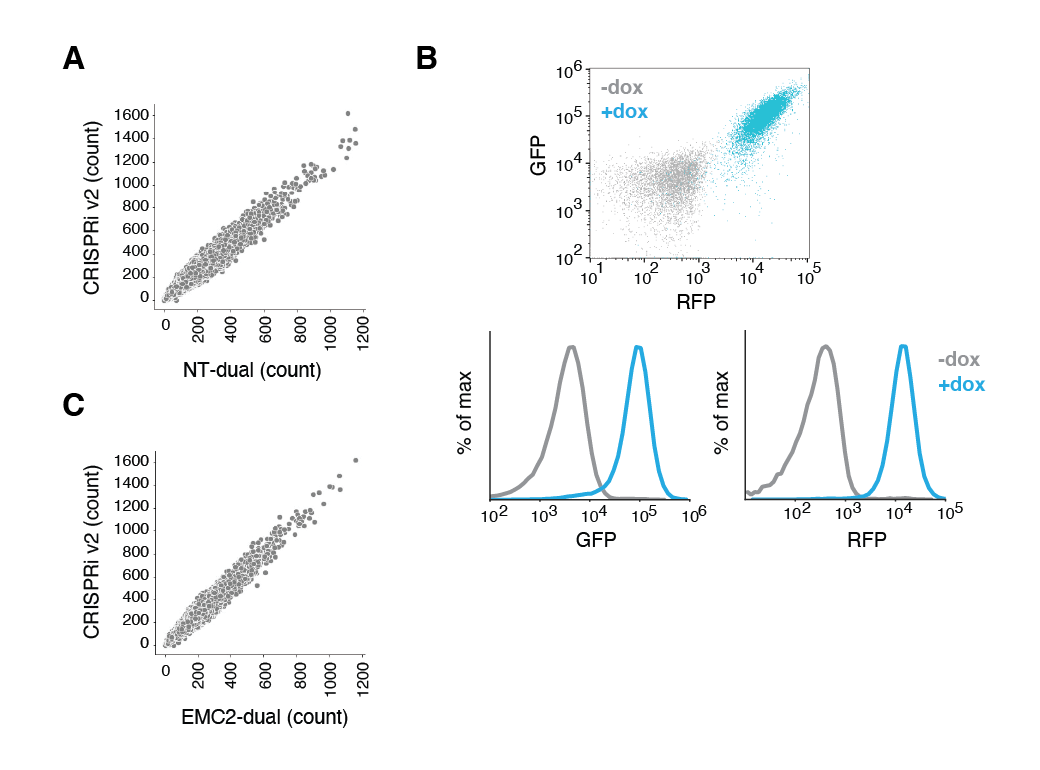
**

**Figure S1. Dual library guide coverage and reporter line characterization.** **(A)** Coverage of genome-wide guides in the NT dual library. Comparison of guide counts from the single CRISPRi-v2 library and the NT-dual library, after excluding guides which drop out due to restriction enzyme cutting during library construction. **(B)** K562 cells expressing GFP1-10 in the ER lumen and the SEC61β -GFP11 reporter under an inducible promoter are treated with doxycycline and analyze by flow cytometry. Green and red channels are shown separately. **(C)** As in (A) for the EMC2 dual library.

**
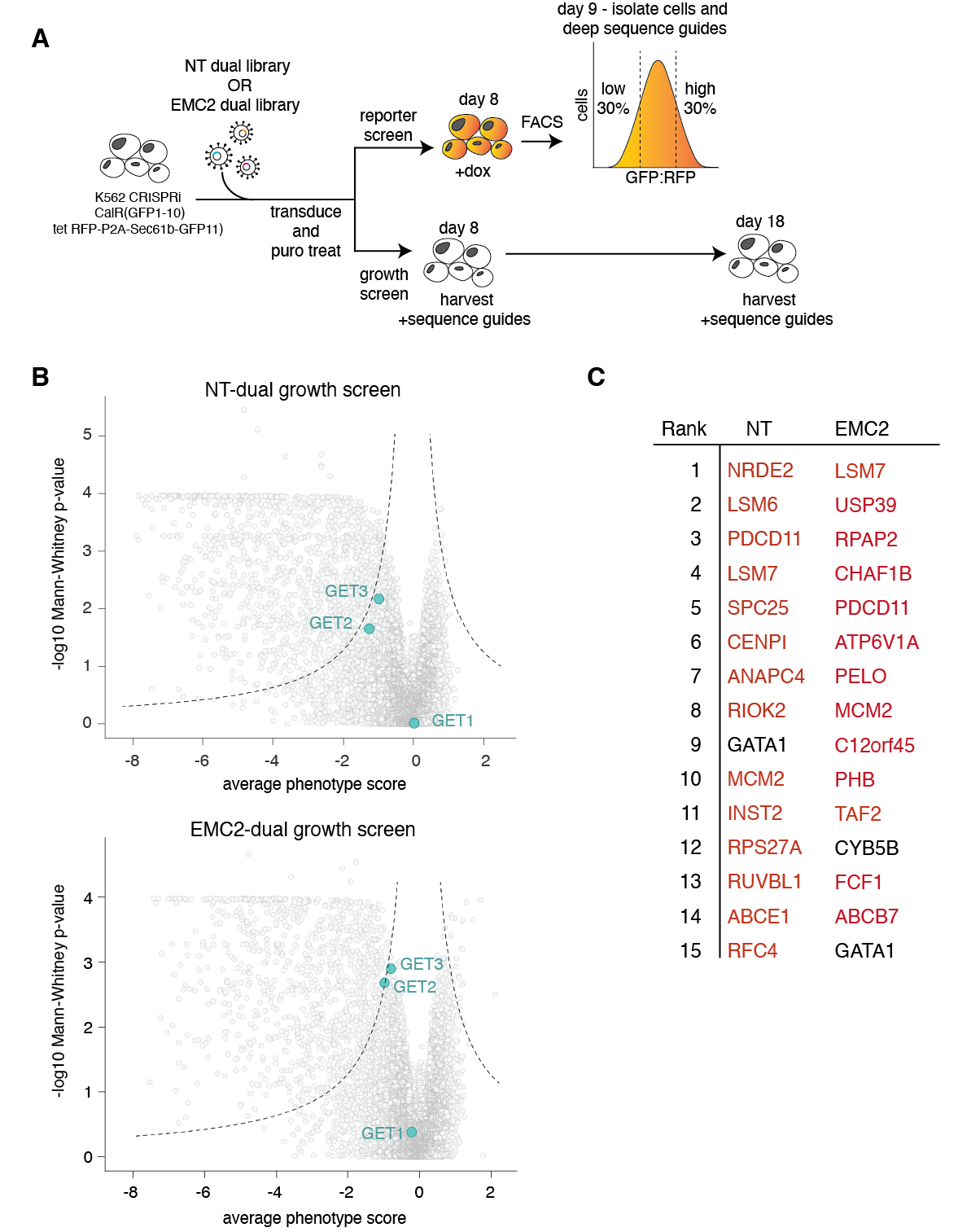
**

**Figure S2**. **Growth screens with dual-guide libraries.** **(A)** Schematic and timeline of CRISPRi fluorescent and growth screens with dual-guide libraries. **(B)** Volcano plots of growth screens for the three strongest guide RNAs versus Mann-Whitney p-values from two independent replicates of growth screens with the indicated libraries. Individual guides are displayed in gray, while core factors of the GET pathway are highlighted in pink. (**C**) Top ranked hits, as measured from discriminant scores, from (B), essential genes are highlighted in red (Tsherniak et al., 2017; Behan et al., 2019).

**
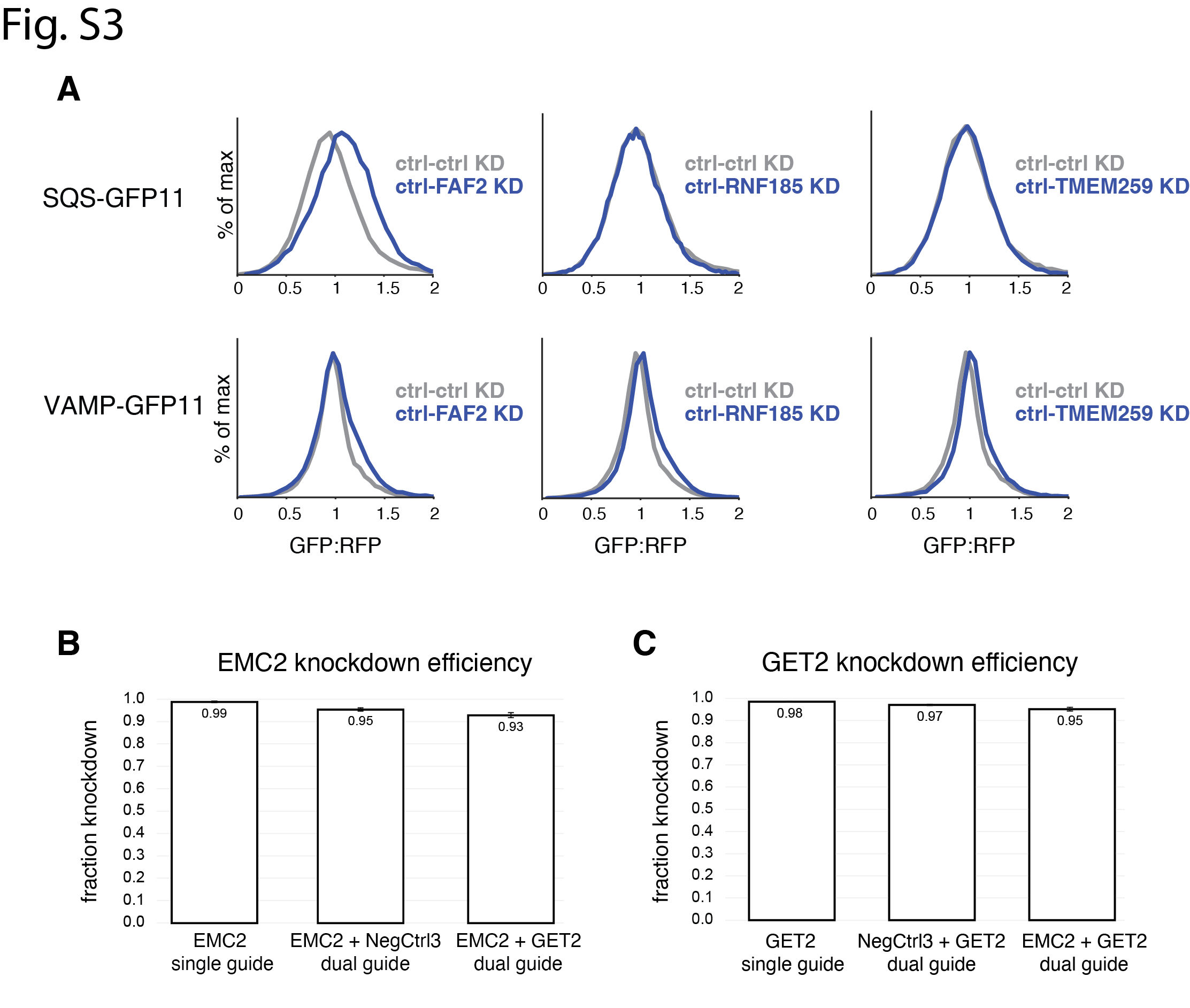
**

**Figure S3**. Investigating the specificity of putative TA quality control factors and assessing knock-down of dual-guides. (A) The stability of SQS-GFP11 and VAMP-GFP11 was assayed as in Figure 4B. (B) qPCR of EMC2 was assessed in K562 CRISPRi cells expressing the indicated guides to determine whether the context of a dual-guide affected knock-down efficiency (normalized to the housekeeping gene HPRT1). See methods for specific details. (C) As in (B) for the GET2 targeting guide.

**Supplementary File Legends**

Supplemental Table 1. Restriction enzyme susceptible guides during dual library construction

Supplemental Table 2. Genome-wide FACS screens with non-targeting and EMC2 dual libraries for TA insertion at the ER

Supplemental Table 3. Genome-wide growth screens with non-targeting and EMC2 dual libraries for TA insertion at the ER
